# Wounds of the Chest

**Published:** 1918-08

**Authors:** P. B. Soltau


Vol. II, N° 1 PARIS AUGUST 1918

### WAR MEDICINE

Published by the American Red Cross Society in France
for the
Medical Officers of the American Expeditionary Forces

### RESEARCH SOCIETY REPORTS

THE SEVENTH SESSION OF THE RESEARCH SOCIETY OF THE AMERICAN RED CROSS IN FRANCE

June 28 and 29, 1918, at the Hotel Continental, Paris.

Major Walter B. Cannon, Chairman of the Research Committee, presided throughout the session.

The subject of the first meeting, Friday June 28 at 2 : 00 P. M. was " Wounds of the Chest ". It was discussed by Col. A. B. Saltau, Captain Lockwood, Sir John Rose Bradford, Dr. Duval, Dr. Tuffier and Dr. Petit de Villéon.

On Saturday, June 29, the subject of War Psychoneuroses was discussed by Major Foster Kennedy, Professor Dupré, Professor Roussy, Professor Laignel-Lavastine, Lt.-Colonel Salmon, Major Rheim and others. Resumés of these papers and discussions are published in this number of WAR MEDICINE.

### WOUNDS OF THE CHEST

**Wounds of the Chest.** Colonel P. B. SOLTAU, R. A. M. C., said that the chest wound was the meeting ground for the physician and the surgeon.

*Incidence.* The chest wound formed on an average 2.5 o/o of all casualties admitted to the casualty clearing stations and, on occasions, it had risen as 3.5 o/o. To deal properly with cases in these numbers he considered that the following preparations were necessary in a casualty clearing station :

A chest team, consisting of a physician and surgeon, who should work in close collaboration, selecting the right type of case for operation, etc.

A ward with 50 beds, of which 20 should be fitted with the Fowler elevating frame. (This would allow for the accommoda[illegible]on

ARMY MEDICAL LIBRARY
Sept. 21 35
31645
WASHINGTON, D. C.

WI
WA2592
v.2
1918-
1919

and retention of cases admitted during 48 hours, on an estimated basis of 1000 admissions a day, which might be expected in active times.)

A skilled nursing staff and trained orderlies; apparatus for giving oxygen, preferably Haldane's; if possible, an operating theater attached; immediate access to a bacteriological laboratory and an X-ray plant. It was necessary also to have a systematized method of note-taking and recording, such as that published by the Medical Research Committee.

*Mortality.* This had been calculated from returns made from the greater part of the British front during the fighting in 1917. It was impossible to be certain of the death rate in the regimental aid post, but such deaths were practically all in the fatally wounded.

At the field ambulance stations it was 7 o/o.

In the casualty clearing stations it was 17.18 o/o, i. e. 15.9 o/o in the 93 remaining cases.

At the Base it was about 6 o/o, i. e. 4.6 o/o in the 77 remaining cases. The total mortality in 100 cases which reached a medical unit was therefore 27.5 o/o.

*Causes of Death.* These were two-fold — (a) anatomical, (b) septic.

*a*) This class included gross lesions, with profound shock and hemorrhage, the complicating lesions involving abdomen and other areas, the multiple wounds, and deaths from edema of the lung and asphyxia.

*b*) The types of infecting organisms had been worked out by Col. Elliot at the Base as follows — gas forming organisms, 48 o/o; streptococci, 40 o/o; and lung organisms 12 o/o. The infected cases in the front area showed much the same distribution of organisms. The streptococcic infections were the most fatal.

In the field ambulance zone the death rate was due entirely to anatomical causes; in the casualty clearing stations it was due both to anatomical and to septic causes, whilst at the base it was entirely septic in origin.

The septic death rate in the casualty clearing stations area was about 26 o/o of all deaths. At one battle it worked out in the proportion of 1 to 3. Gask and Wilkinson found in their series that sepsis caused 31 o/o of the death rate. Applying the figures given as to mortality, it will be seen that of every 100 men wounded in the chest, about 9 die of sepsis. This is the death rate that surgery has the greatest hope of reducing.

*Types of Wound.* These were classified as tangential, penetrating (or briefly as " E ", entrance wound) and perforating (or " E " and " E ", entrance and exit wounds). The tangential wound, involving muscle and bone and yet not penetrating the pleura often caused, nevertheless, an intra-thoracic injury — hemothorax, infarction of lung, etc.

The character of the missile was naturally responsible for a great variety of wounds. Large shell fragments would smash many ribs, lacerate the parieties extensively, and destroy a large area of lung. Shell fragments of whatever size were more likely to be retained inside the thorax than bullets. Bullet wounds might also vary greatly in degree. A perforating bullet might traverse the lung and yet cause no discoverable lesion. At times, on the other hand, most extensive injuries were seen. This was due partly to the oscillating movement of the bullet which, in addition to its forward movement and its rotation round the long axis, imparted by the rifling, had potential rotation round its short axis, most marked at the early and late stages of the flight, and made active whenever the missile encountered resistance. This short axis rotation was responsible for many of the so-called " explosive " effects. When it was remembered that the Mauser bullet had, at muzzle velocity, a striking force equal to over 30 foot tons, the extent of the damage caused would be realized.

*Nature of Injuries.* (*a*) To Parieties. All degrees of muscle and bone injury are met with. As elsewhere in the body, these required treatment surgically, as soon as the patient was in a fit state for operation. It must not be forgotten that the muscles of the shoulder girdle were liable to gas infection, and wounds in that area needed most careful watching. The " stove-in " chest was a term applied to a chest in which many ribs had been fractured and fragments had been driven into the pleura. This formed a most dangerous wound, and one which until recently had been nearly always fatal. Surgery, skilfully performed, would succeed in a certain number of these cases, and, in such a desperate condition, operative measures were justified as constituting practically the only hope, for even if the patient survived the initial shock, fatal sepsis nearly always supervened.

(*b*) To Lung. The conditions set up by injury to the lung were as follows : laceration, hemothorax, and collapse, either singly or in combination. It was impossible in the time available to enter into any discussion as to the physical signs produced by these varying conditions, and time and experience were needed to elucidate the often baffling physical signs. Civil experience of the physical signs in the chest must be largely abandoned.

As regards the lung injury itself, the most common condition was infarction along the track. Happily sepsis in the wounded lung was not very frequent, as that organ had a remarkable power to deal with infection within its substance.

Both hemothorax and pneumothorax might be derived from external and from pulmonary sources. It was probable that in the majority of cases the blood was poured into the pleural cavity from the lung and not from torn intercostal vessels.

Collapse of the lung was an almost invariable condition, the mechanism of the production was as yet little understood. It appeared to be an active process, and might even be regarded as Nature's method of causing hemostasis and checking the escape of air. A discussion of the mechanism would be impossible in the time available.

One point as regards pneumothorax required emphasis, and that was the condition known as the sucking wound, where air was being drawn in and out of the pleural cavity through the wound. This was a most dangerous condition causing profound shock owing to the constant variation of intra-thoracic pressures, and oscillation of the mediastinum, and rendering the patient very prone to sepsis. Since the practice of closing such a wound at the earliest possible opportunity had been introduced, a great improvement in results had been seen. Nothing was more dramatic than the almost instant relief given by such a closure, which could frequently be done by a couple of skin stitches.

*Contralateral Conditions.* Particular attention must always be paid to the condition of the unwounded lung, on which so largely depended the fate of the patient. Frequently there was observed an area of collapse, " contralateral collapse ", which varied in degree from a partial deflation to a massive collapse of the lower lobe. Contralateral bronchitis and edema were also very frequent. Stress was laid on these conditions, as their existence was a bar to operation.

*Shock.* The chest wound was liable to be accompanied by profound shock. Without introducing the controversial subject of acidosis in shock, it might be pointed out that because of the resulting pain, exhaustion, diminished oxygenation from hemorrhage and from loss of ventilating area, forced respiratory movements and impeded circulation, the chest wound was pre-eminently the wound in which the production of acidosis was favored.

*Treatment.* This huge subject could be dealt with only superficially.

In the line, the following points required attention : cleanliness; the combating of shock by careful warming, taking care not to overdo the " réchauffement "; the relief of pain by morphia or one of its derivatives, such as heroin or omnopon, and by placing the patient in the position which he himself found most confortable. It must always be remembered that the chest wound is accompanied by psychical disturbance, for nothing so frightens a man as difficulty in breathing, and possibly the sound of air whistling through an open wound. The necessity of closing an open wound must again be mentioned, this is best done by suture, and, where this is impossible, by most careful plugging. The case should be evacuated to a casualty clearing station as soon as the primary shock is overcome, usually in about three or four hours.

*At the casualty clearing station :*

The first essential is to secure rest. Early examination should be confined to determining that there is neither bleeding nor a sucking wound. More detailed examination may follow in a few hours, when an exact diagnosis as to the conditions present, coupled with X-Ray localization of foreign bodies, etc., should be made.

Sepsis must be continually watched for, both by needling and by observing the constitutional signs. The large hemothorax needs aspiration, which is best done about 48 to 72 hours after wounding. As regards surgery, the following types of wound might be taken as needing operation : the large open wound or the " stove-in " chest, requiring plastic repair, and closure; the large retained foreign body, when accurately localized; the infected chest, which could be treated either by cleansing and closure or by drainage.

*When to Evacuate.* This is a constant problem especially in times of active fighting. Only certain general indications can be given. In quiet times it is better to evacuate the straightforward cases either at the end of three days or else after ten days. The intervening period is the time when sepsis usually appears, and it is better not to let cases travel during that period.

In rush times, all wounds unaccompanied by extensive parietal injury, in which the hemothorax is not excessive, may be evacuated within 24 hours, provided the primary shock is overcome.

After operation, cases should be retained for at least 10 days.

The open or draining chests travel badly.

Cases should not be evacuated within 24 hours of aspiration.

Colonel Soltau said that it was better to hold all cases until convalescent, but the ideal was rarely attainable, and it was often absolutely essential to evacuate early. The data he had given were to enable officers to select the cases best able to travel. He wished to emphasize that surgical treatment concerned only a small proportion of the cases, that in these the highest skill was necessary, and that for the remainder the painstaking care of a skilled physician was needed.

